# 617. The Impact of Burn Injury on Psychiatric Outcomes in Pediatric versus Adult Patients

**DOI:** 10.1093/jbcr/irag033.431

**Published:** 2026-04-06

**Authors:** Philong Nguyen, Aidan R Carroll, Joshua Wang, Luka Zrnic, Noelle Gonzalez, Kamryn K Baker, Sudhanvan Iyer, Cameron Bowers, Yousef Tanas, Jong Lee

**Affiliations:** University of Texas Medical Branch, Galveston, TX; University of Texas Medical Branch, Galveston, TX; University of Texas Medical Branch, Galveston, TX; University of Texas Medical Branch, Galveston, TX; University of Texas Medical Branch - John Sealy School of Medicine, Galveston, TX; McGovern Medical School at University of Texas Health Science Center at Houston, Houston, TX; University of Texas Medical Branch, Galveston, TX; University of Texas Medical Branch, Galveston, TX; Houston Methodist Hospital, Houston, TX; Division of Burn and Trauma Surgery, Department of Surgery, University of Texas Medical Branch, Galveston, Galveston, TX

## Abstract

**Introduction:**

Burn injuries are frequently followed by profound psychological consequences, including depression, anxiety, PTSD, and diminished quality of life. Both children and adults face mental health burdens after burns, but age-specific risks remain poorly defined. To date, no large-scale study has directly compared psychiatric outcomes between pediatric and adult patients. This retrospective cohort study uses a large multicenter database to compare psychiatric outcomes in pediatric versus adult burn patients.

**Methods:**

The TriNetX Research Network was queried for patients who sustained burn injuries between 2002 and 2022. Two age groups were defined using ICD-10 codes: pediatric patients (< 18 years) and adult patients (≥18 years). Propensity score matching (1:1) was performed within each cohort based on demographics, socioeconomic factors, total burn surface area, and burn region. Primary outcomes included new diagnoses of anxiety, depression, post-traumatic stress disorder (PTSD), adjustment disorder, suicidal ideation/self-harm, substance use disorders (SUD), body dysmorphic disorder, and sleep disorders. Secondary outcomes included new antidepressant use. Patients with prior psychiatric diagnoses or antidepressant use before the study window were excluded. Outcomes were assessed at 3 months, 1 year, and 3 years, with statistical significance set at *p*<.05.

**Results:**

After matching, 206 789 patients were included in each cohort. At 3 months, pediatric burn patients had significantly lower incidence of anxiety (0.74% vs 2.34%, HR 0.32, *p*<.001), depression (0.24% vs 1.49%, HR 0.16, *p*<.001), suicidal ideation/self-harm (0.11% vs 0.27%, HR 0.40, *p*<.001), PTSD (0.12% vs 0.38%, HR 0.30, *p*<.001), sleep disorders (0.36% vs 1.27%, HR 0.28, *p*<.001), substance use disorder (0.17% vs 2.30%, HR 0.07, *p*<.001), adjustment disorder (0.22% vs 0.42%, HR 0.54, *p*<.001), and antidepressant use (0.96% vs 3.36%, HR 0.31, *p*<.001) compared with adults. Body dysmorphic disorder showed no significant differences at any time point. These trends remained consistent at 1 and 3 years, with previously observed reductions persisting and no new significant associations emerging.

**Conclusions:**

Pediatric burn patients experienced lower psychiatric morbidity compared to adults, with differences persisting for at least three years. These findings underscore the importance of age-specific mental health screening and support following burn injury. Prospective studies are needed to validate these results and elucidate mechanisms underlying psychiatric differences across age groups.

**Applicability of Research to Practice:**

Recognizing the distinct psychiatric outcomes of pediatric and adult burn survivors supports proactive, age-specific mental health screening, multidisciplinary care, and early resource allocation to optimize recovery and long-term quality of life.

**Funding for the study:**

This study was supported by foundation funding through the Institute for Translational Sciences (UL1 TR001439), funded by the National Center for Advancing Translational Sciences at the NIH.

